# Supplementary material for: Molecular asymmetry of a photosynthetic supercomplex from green sulfur bacteria
Source: Nat Commun. 2022 Oct 3;13:5824. doi: 10.1038/s41467-022-33505-4 (PMC9529944; doi:10.1038/s41467-022-33505-4)
Supplement: Supplementary file 1 — Supplementary Information [file 41467_2022_33505_MOESM1_ESM.pdf]

SUPPLEMENTARY INFORMATION FOR

Molecular asymmetry of photosynthetic supercomplex from green sulfur bacteria

Ryan Puskar[1,2], Chloe Du Truong[1,2†], Kyle Swain[3], Saborni Chowdhury[1,2], Ka-Yi Chan[1,2], Shan Li[4], Kai-Wen Cheng[4], Ting Yu Wang[5], Yu-Ping Poh[2‡], Yuval Mazor[1,2], Haijun Liu[6], Tsui-Fen Chou[4,5], Brent L. Nannenga[2,3], Po-Lin Chiu[1,2]*

[1]School of Molecular Sciences, Arizona State University, Tempe, AZ 85287

[2]Center for Applied Structural Discovery, Biodesign Institute, Arizona State University, Tempe, AZ 85287

[3]School for Engineering of Matter, Transport and Energy, Arizona State University, Tempe, AZ 85287

[4]Division of Biology and Biological Engineering, California Institute of Technology, Pasadena, CA 91125

[5]Proteome Exploration Laboratory, Beckman Institute, California Institute of Technology, Pasadena, CA 91125

[6]Department of Biology, Washington University, St. Louis, MO 63130

[†]Present address: Rampart Bioscience, Monrovia, CA 91016

[‡]Present address: Center for Mechanisms of Evolution, Biodesign Institute, Arizona State University, Tempe, AZ 85287

*Corresponding author: Po-Lin Chiu (plchiu@asu.edu)

SUPPLEMENTARY FIGURES

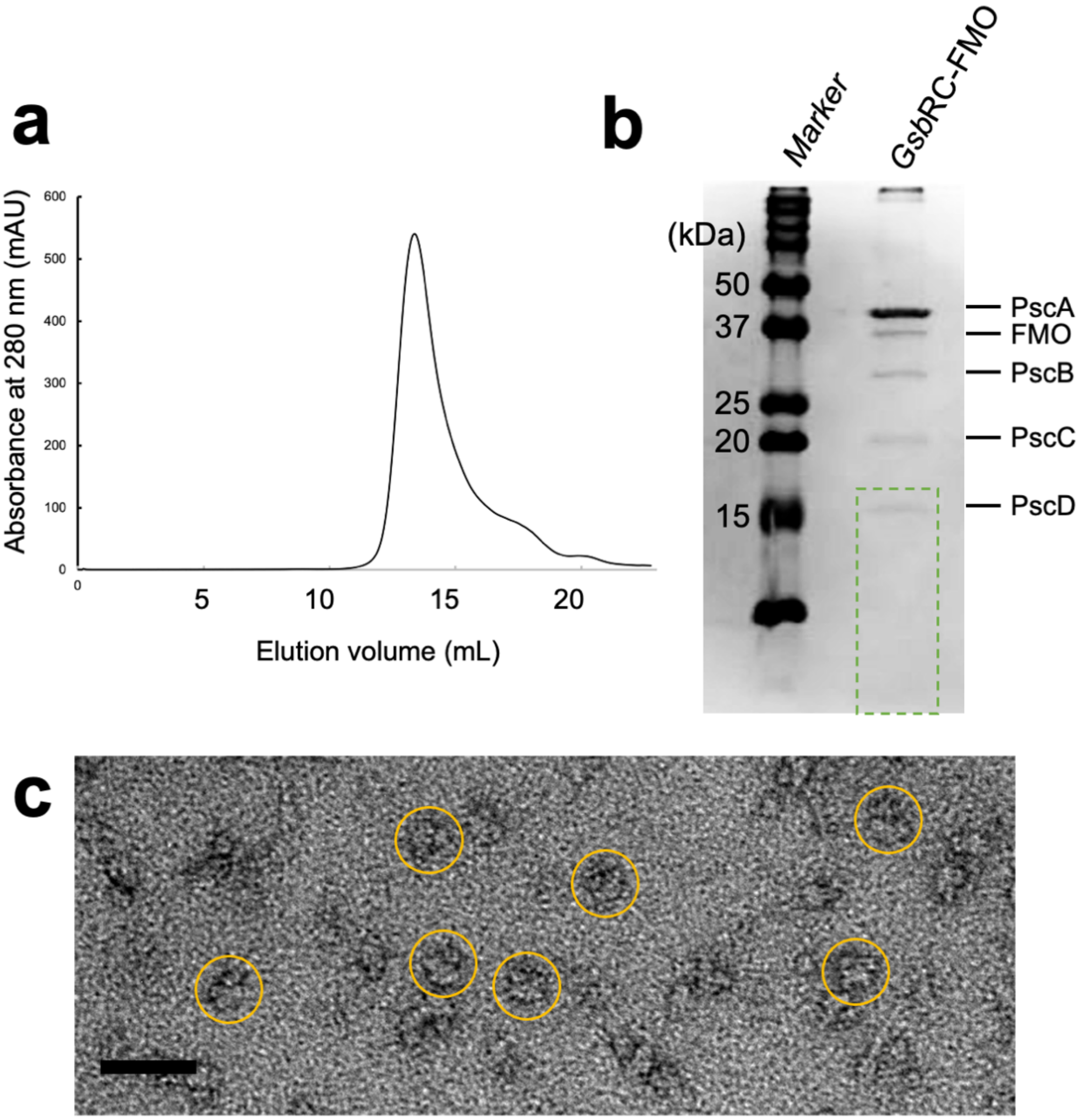

Supplementary Fig. 1 | Characterization of membrane extraction from *Chlorobaculum tepidum*. a, Size-exclusion chromatographic (SEC) profile of the detergent-solubilized sample. **b,** SDS-PAGE of the eluted peak fraction from the SEC. The reaction center (PscA, PscB, PscC, and PscD) and FMO complexes of *C. tepidum* are shortened as GsbRC-FMO. Gel bands within the highlighted green rectangle (< 15 kDa) were excised and digested for further mass spectrometry analysis. The results were reproducible for more than three times. **c,** Representative electron micrograph (*n* > 10) of the negatively stained protein sample. Representative particles for the target protein complexes are circled in orange. Scale bar indicates 60 nm.

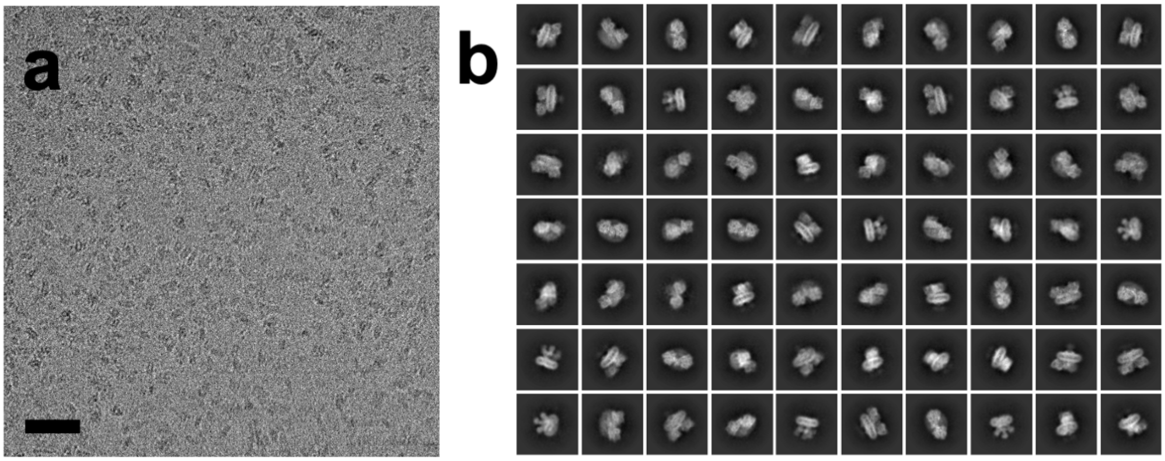

Supplementary Fig. 2 | Cryo-EM image analysis of membrane protein complexes from *C. tepidum*. a, Representative electron image of cryogenic protein complexes from collected 32,898 micrographs. Black contrasts are particles of protein complexes. Scale bar indicates 50 nm. **b,** Representative 2D class averages of the single-particle images. Box side length is 381 Å.

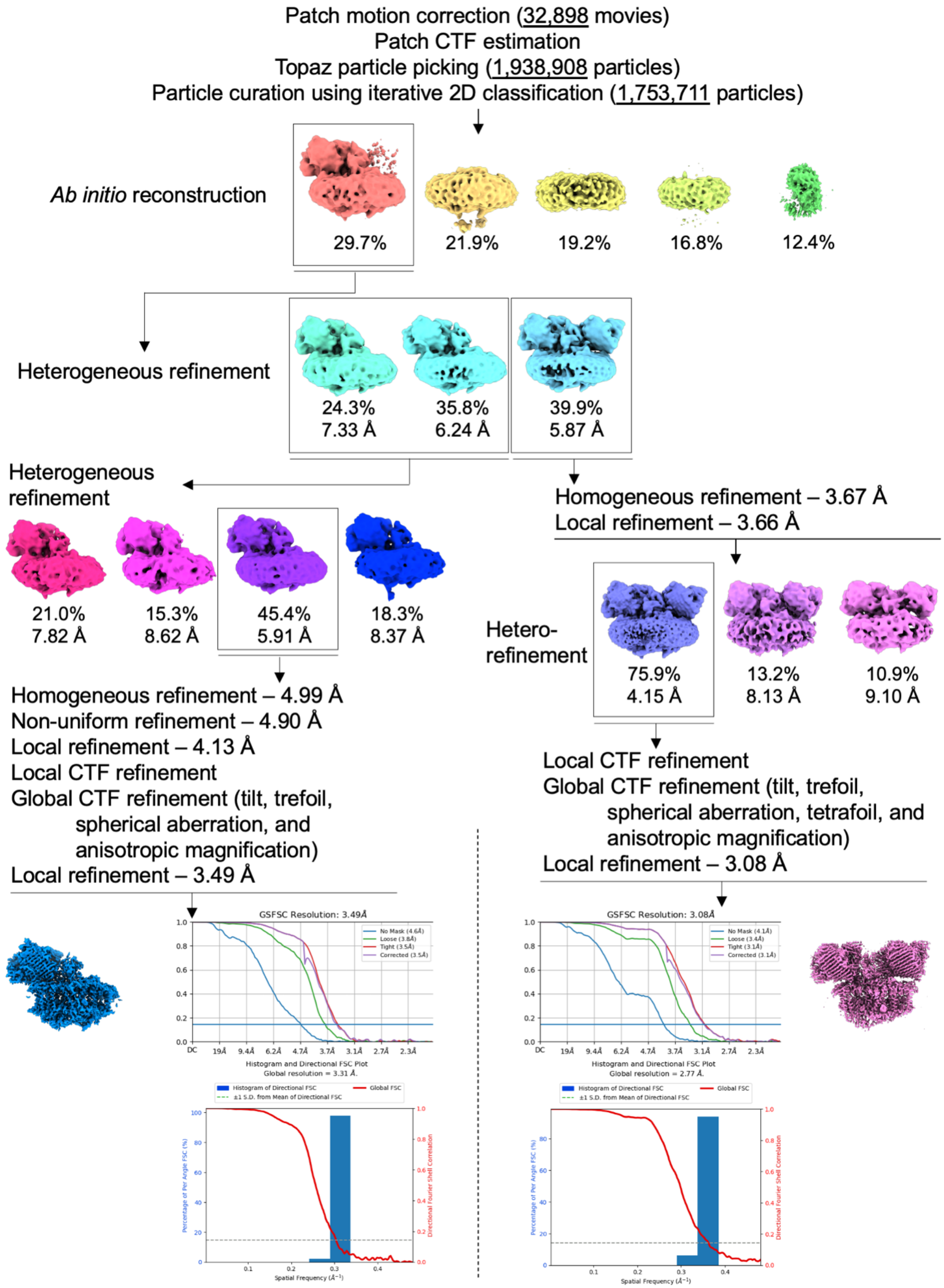
Patch motion correction (32,898 movies)
Patch CTF estimation
Topaz particle picking (1,938,908 particles)
Particle curation using iterative 2D classification (1,753,711 particles)
Ab initio reconstruction
29.7%
21.9%
19.2%
16.8%
12.4%
Heterogeneous refinement
24.3%
7.33 Å
35.8%
6.24 Å
39.9%
5.87 Å
Heterogeneous refinement
21.0%
7.82 Å
15.3%
8.62 Å
45.4%
5.91 Å
18.3%
8.37 Å
Homogeneous refinement – 4.99 Å
Non-uniform refinement – 4.90 Å
Local refinement – 4.13 Å
Local CTF refinement
Global CTF refinement (tilt, trefoil, spherical aberration, and anisotropic magnification)
Local refinement – 3.49 Å
Homogeneous refinement – 3.67 Å
Local refinement – 3.66 Å
Hetero-refinement
75.9%
4.15 Å
13.2%
8.13 Å
10.9%
9.10 Å
Local CTF refinement
Global CTF refinement (tilt, trefoil, spherical aberration, tetrafoil, and anisotropic magnification)
Local refinement – 3.08 Å
GSFSC Resolution: 3.49Å
No Mask (4.6Å)
Loose (3.8Å)
Tight (3.5Å)
Corrected (3.5Å)
Histogram and Directional FSC Plot
Global resolution = 3.31 Å.
Histogram of Directional FSC
Global FSC
±1 S.D. from Mean of Directional FSC
Percentage of Per Angle FSC (%)
Directional Fourier Shell Correlation
Spatial Frequency (Å⁻¹)
GSFSC Resolution: 3.08Å
No Mask (4.1Å)
Loose (3.4Å)
Tight (3.1Å)
Corrected (3.1Å)
Histogram and Directional FSC Plot
Global resolution = 2.77 Å.
Histogram of Directional FSC
Global FSC
±1 S.D. from Mean of Directional FSC
Percentage of Per Angle FSC (%)
Directional Fourier Shell Correlation
Spatial Frequency (Å⁻¹)

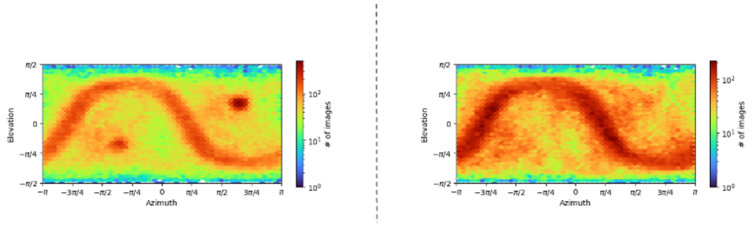

Supplementary Fig. 3 | Single-particle reconstruction of the GsbRC-FMO complex. Image processing flowchart on single-particle cryo-EM data of the protein complexes from *Chlorobaculum tepidum*. Golden Fourier-shell correlation (FSC), directional FSC plots, and angular distributions for individual reconstructions are shown in the lower panel.

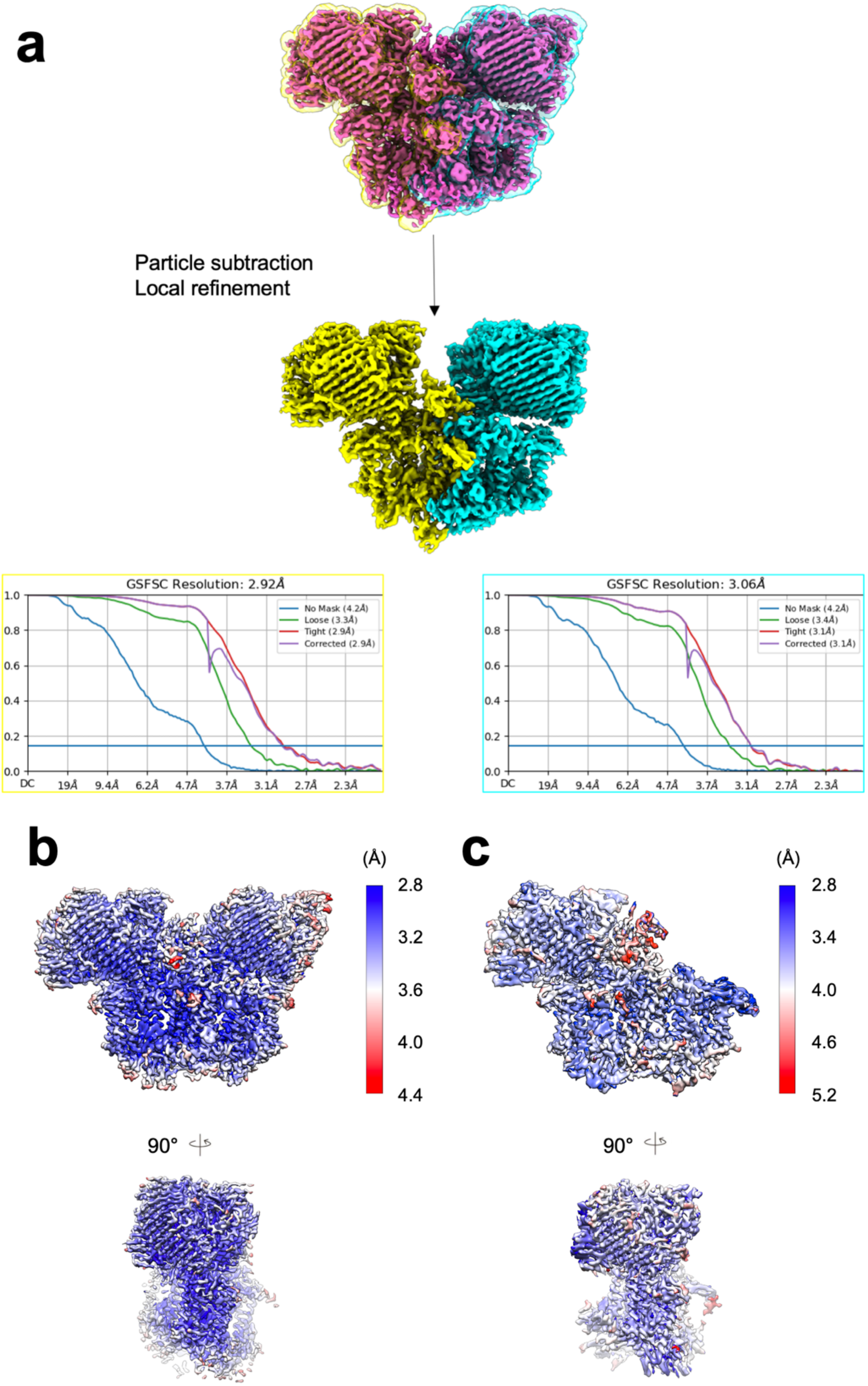

Supplementary Fig. 4 | Signal subtraction, local refinement, and local resolution estimation on the cryo-EM densities of the RC-FMO assemblies. a. Overall density of the RC-FMO_2

assembly is shown in pink. Masks used for local refinement are shown in light blue (FMO1, PscA1, and PscB) and light yellow (FMO2 and PscA2) surfaces. Golden Fourier-shell correlation (FSC) plots are shown in the lower panel. Local resolution maps for **b,** RC-FMO_2 and **c,** RC-FMO_1 assemblies. Color bars are presented to indicate the local resolution values of the density maps. Resolutions from low to high are presented in red to blue.

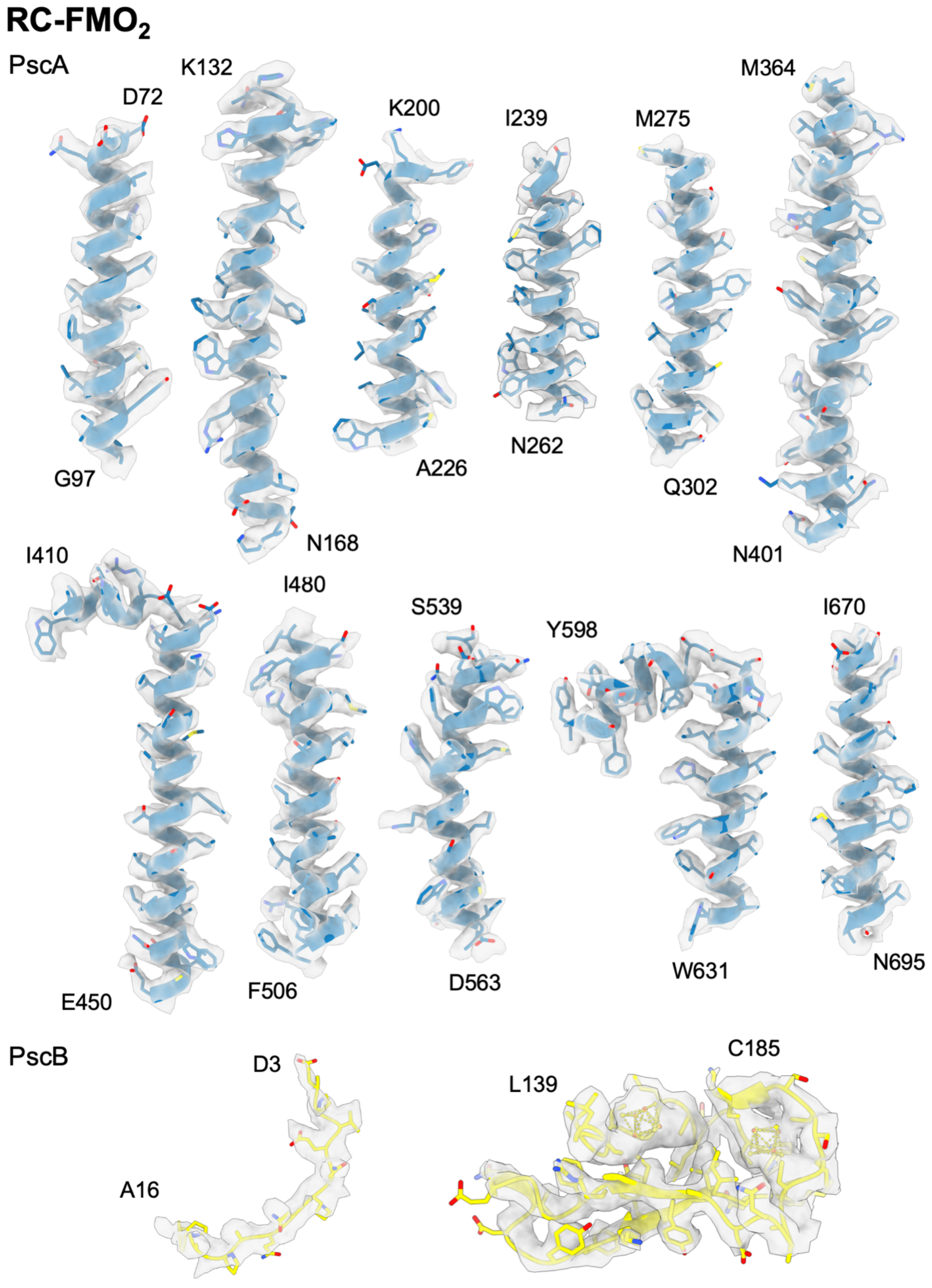
RC-FMO2
PscA
D72
G97
K132
N168
K200
A226
I239
N262
M275
Q302
M364
N401
I410
E450
I480
F506
S539
D563
Y598
W631
I670
N695
PscB
D3
A16
L139
C185

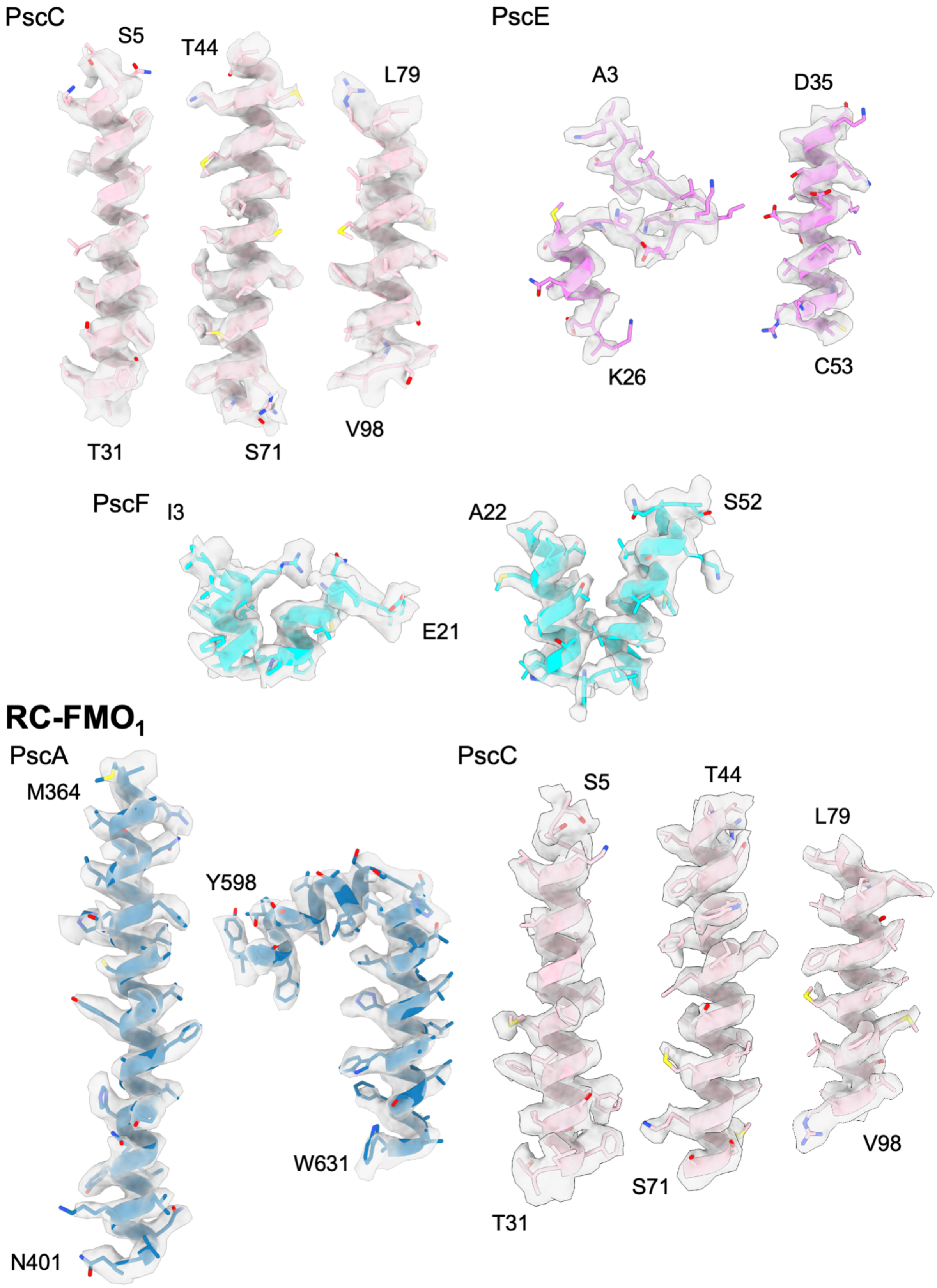
PscC
S5
T44
L79
T31
S71
V98
PscE
A3
D35
K26
C53
PscF
I3
E21
A22
S52
RC-FMO$_1$
PscA
M364
Y598
W631
N401
PscC
S5
T44
L79
T31
S71
V98

Supplementary Fig. 5 | Representative model fitting of the cryo-EM densities of the RC-FMO_2 and RC-FMO_1 complex assemblies. Color codes: PscA1 – blue; PscA2 – light blue; PscB – yellow; PscC – light pink; PscD – purple; PscE – magenta; and PscF – cyan. Grey surfaces present the corresponding cryo-EM densities.

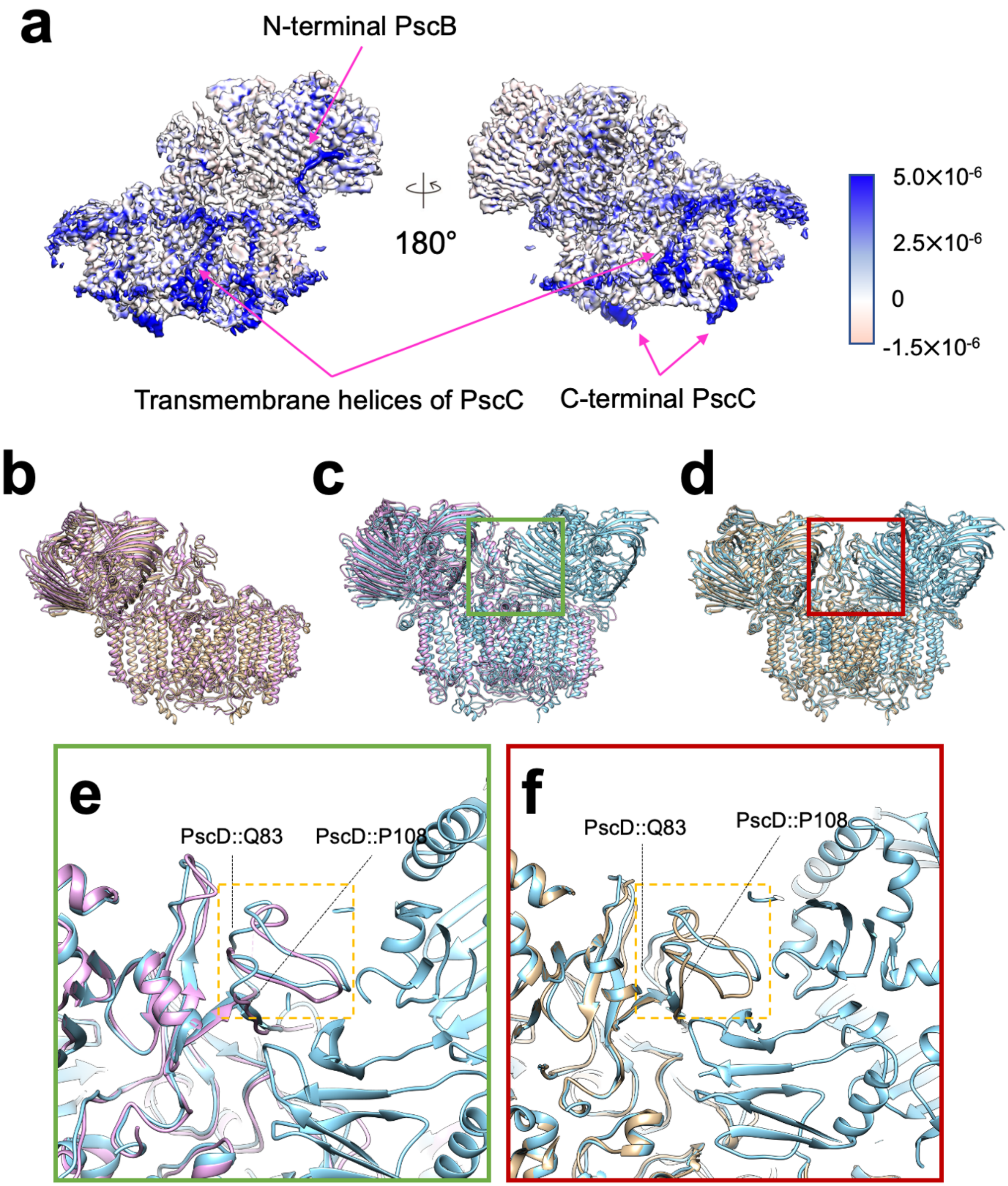

Supplementary Fig. 6 | Structural superpositions of the RC-FMO$_2$ and RC-FMO$_1$ assemblies. a, Density difference between the RC-FMO$_1$ and the previous RC-FMO$_1$ cryo-EM densities (EMD-30069). Subtracted values are shown in blue and red for positive and negative values, respectively, on the map surface. Superpositions of the structures: **b,** RC-FMO$_1$ and 6M32 (RMSD 1.060 Å); **c,** RC-FMO$_2$ and 6M32 (RMSD 1.123 Å); and **d,** RC-FMO$_2$ and RC-

FMO_1 (RMSD 0.777 Å). RC-FMO_2, RC-FMO_1, and 6M32 are colored in light blue, fawn, and pink, respectively. **e** and **f,** Enlarged views of the local regions squared in **c** (green) and **d** (dark red), respectively. FMO2 binding induces a slight loop conformational change on PscD (Q83-P108) (dashed orange square).

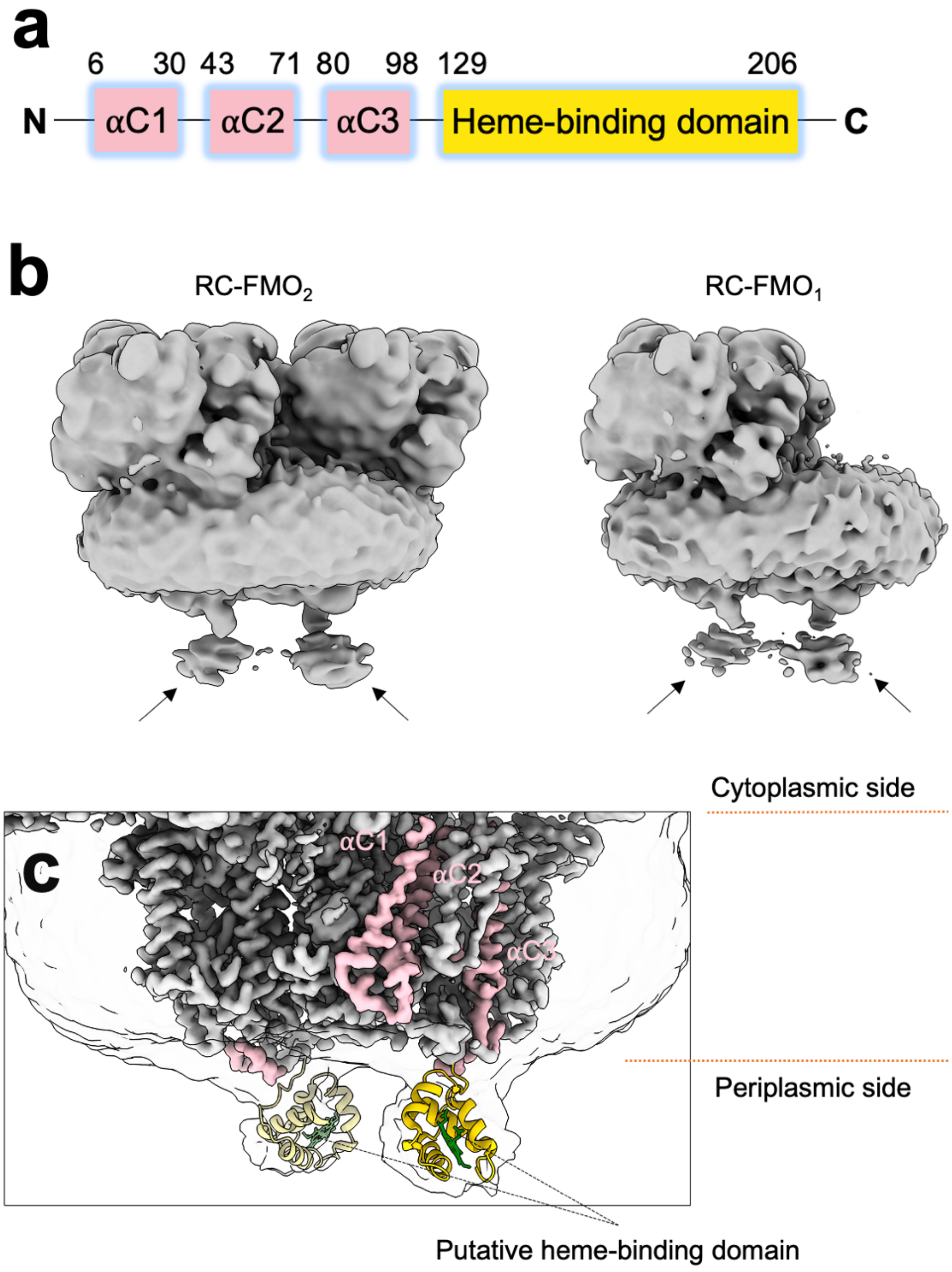

Supplementary Fig. 7 | Structure of the PscC subunit. a, Domain structure of the PscC subunit. **b,** Low-contour cryo-EM densities for RC-FMO$_2$ (1.4σ) and RC-FMO$_1$ (1.8σ). Arrows indicate possible C-terminal heme-binding domains of PscC. **c,** Cryo-EM density of the PscC (light pink) and docking of the atomic coordinate of the heme-binding domain (PDB code: 3A9F; protein residues – yellow; heme - green). Densities of other regions of the complex assembly are shown in grey (3.6σ). Enclosed surface is the density sigma level at 1.0.

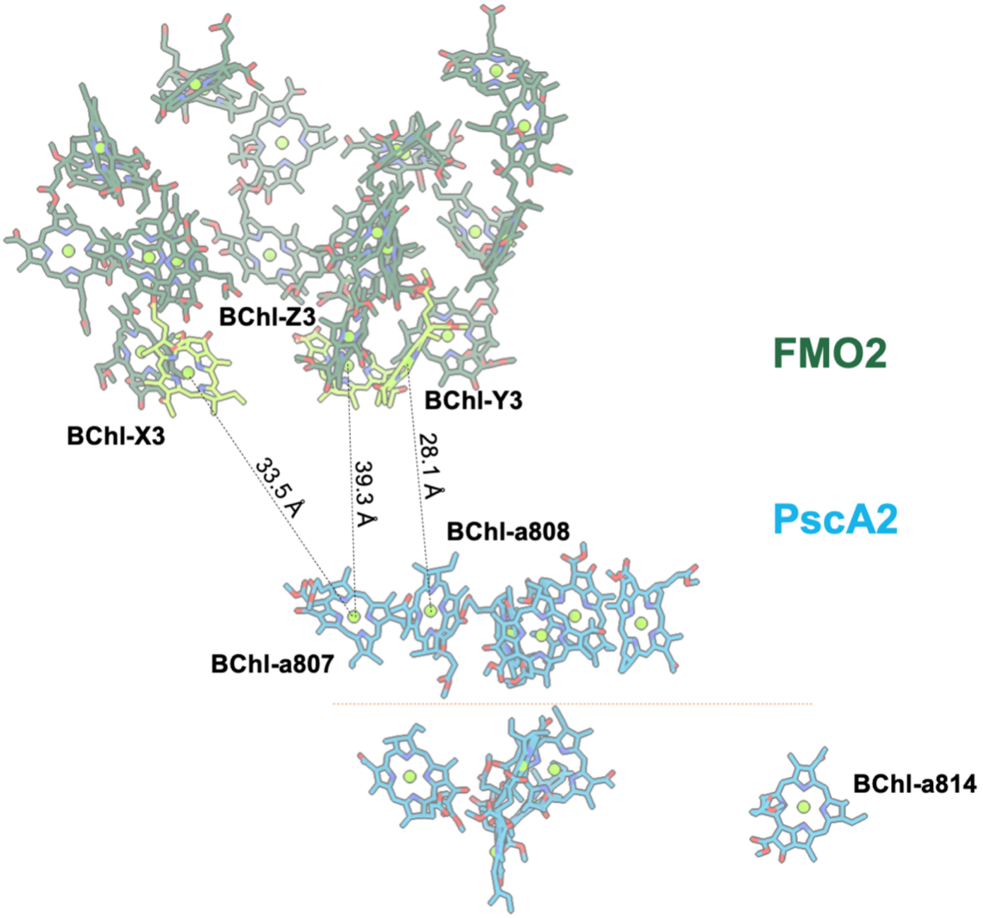

Supplementary Fig. 8 | Distance analyses between pigments along the FMO2-PscA2 axis. The shortest Mg-Mg distance between BChls at the FMO2 (green)-PscA2 (blue) interface is 28.1 Å (BChl-Y3-BChl-a808). Orange dash line separates the BChl clusters of the cytoplasmic and periplasmic layers.

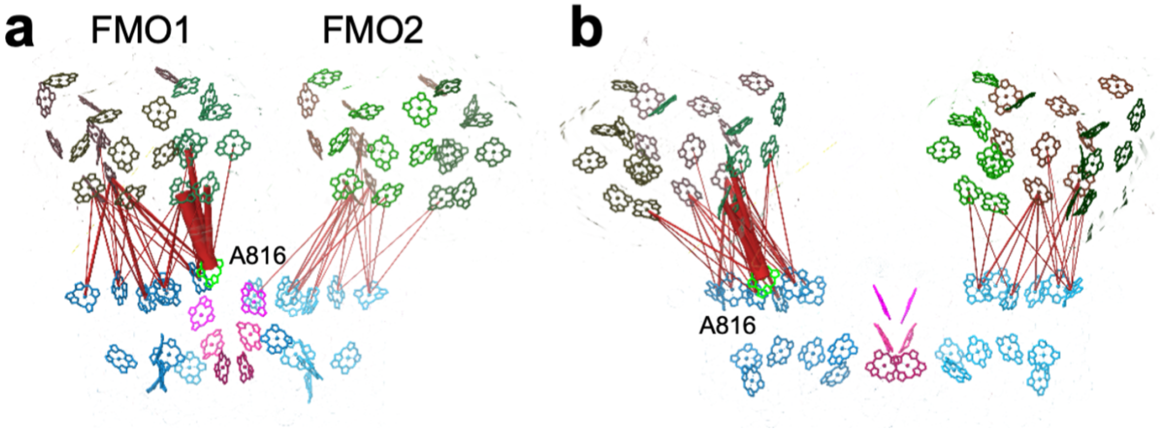

Supplementary Fig. 9 | Energy transfer rates between FMO and RC. a, and **b,** energy transfer rates (calculated using the Förster model) between all the BChls in FMO and RC complexes. Rates were calculated on BChl pairs closer than 40 Å (between FMO and RC) are shown as red lines. The radii of the connecting lines are scaled inversely to the transfer rates. Thicker lines represent faster rates. BChl-A816 alone accounts for more than 50% of the transfer between FMO1 and the RC (FMO1-BChl-A816 alone: 0.07 ps^{-1}; FMO1-RC: 0.11 ps^{-1}). The FMO1 is more efficiently connected to the RC than FMO2 (FMO2-RC: ~0.02 ps^{-1}). Transfer rates and orientation factors for individual BChl pairs are listed in Supplementary Data 1 and 2.

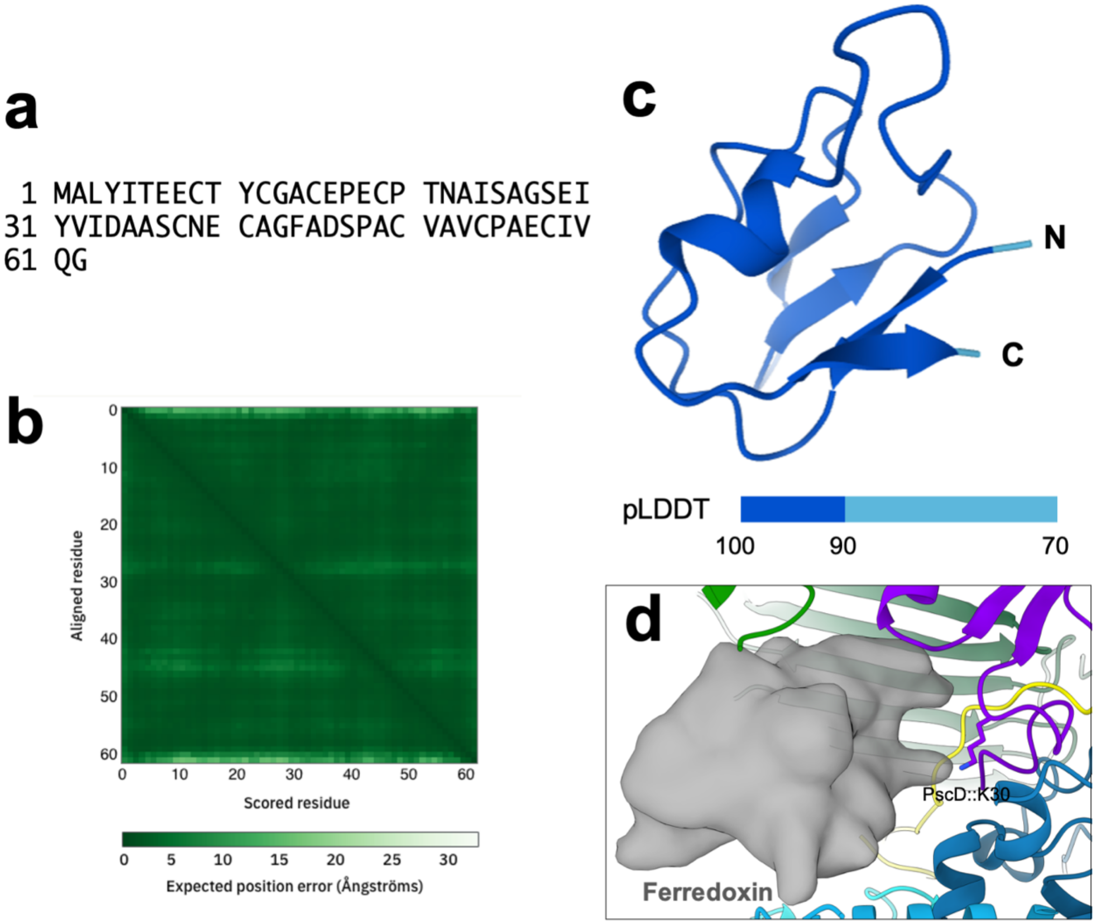

Supplementary Fig. 10 | Association of PscB and ferredoxin in GSB photosynthetic assembly. a, Protein sequence of ferredoxin (accession number: Q8KCZ6). **b,** Predicted aligned error (PAE) plot of the ferredoxin. Graded green to white represents small to large position errors. **c,** Predicted ferredoxin atomic coordinate. Structure is colored by the confidence score from the per-residue local distance difference test (pLDDT). **d,** Potential ferredoxin docking site. Asymmetric binding of the FMO trimers, PscB, and PscD on the RC surface leaves an open space for ferredoxin binding. Ferredoxin is shown in grey surface. A possible lysine residue, K30, of the PscD may involve ferredoxin binding.

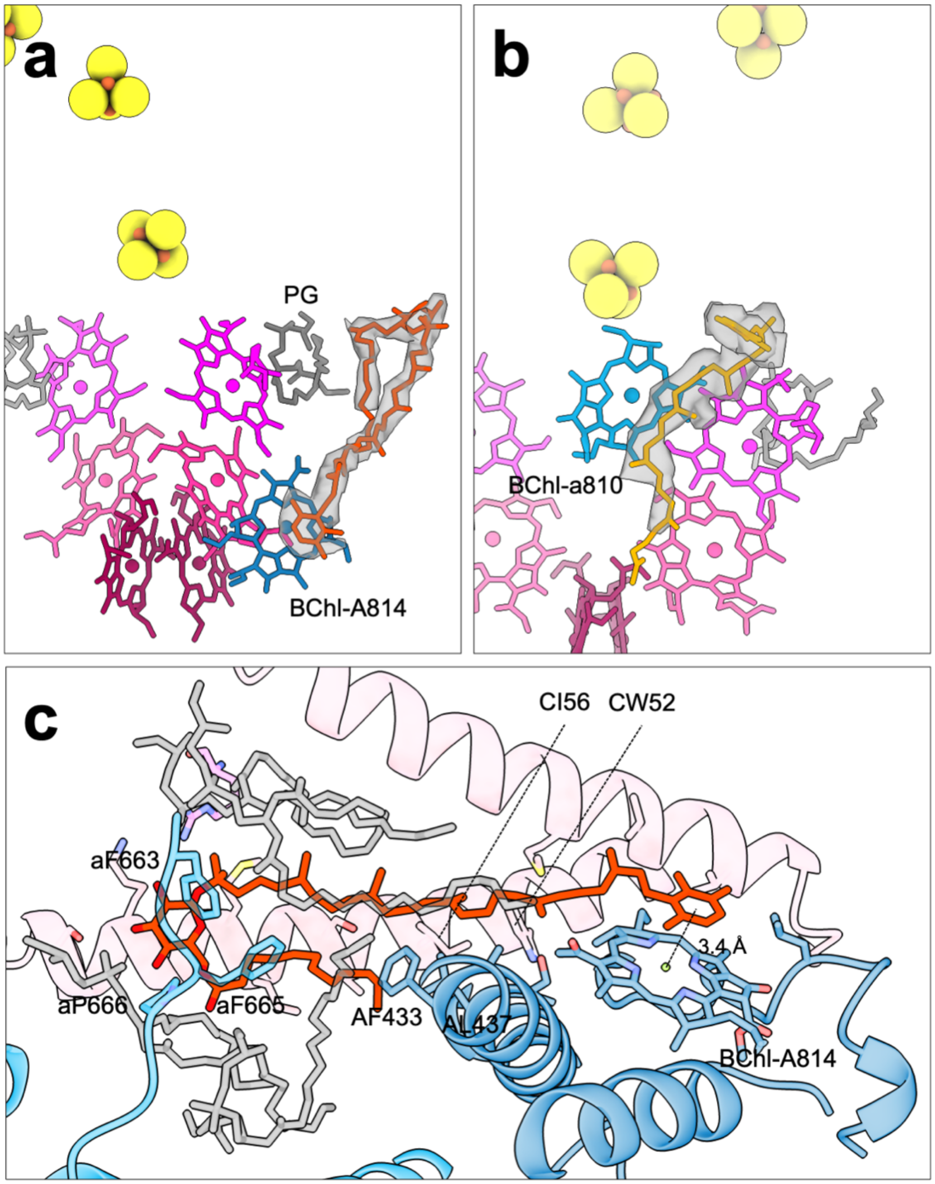

Supplementary Fig. 11 | Newly identified carotenoids in the RC-FMO$_2$ photosynthetic assembly. a, F39 molecule bound in the PscA dimer interface. Cryo-EM density is shown as grey surface (2.1σ). **b,** Additional F26 molecule found in the PscA2 subunit. Cryo-EM density is shown as grey surface (1.5σ). **c,** F39 (orange red) is stabilized by hydrophobic interactions via PscA (light blue), PscC (pink), and lipids (grey). The porphyrin ring of the BChl-A814 interacts with the terminal ring of the F39 at a distance of 3.4 Å.

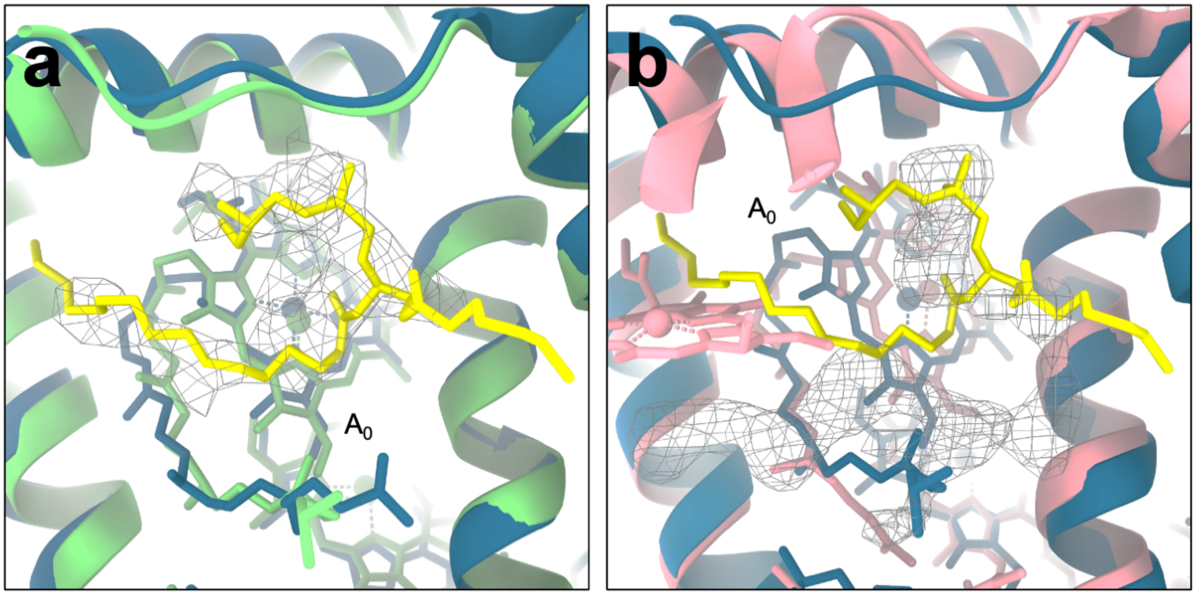

Supplementary Fig. 12 | Unmodelled densities in the previous RC structures from *Chlorobaculum tepidum* and *Heliobacterium modesticaldum*. Structural superpositions of the RC-FMO_2 structure with **a,** the previous RC-FMO_1 structure from *C. tepidum* (PDB code: 6M32) and **b,** the homodimeric RC (*Hb*RC) structure from *H. modesticaldum* (PDB code: 5V8K). RC-FMO_2, the previous RC-FMO_1, and *Hb*RC are colored blue, green, and pink, respectively. The modeled PG lipid in the RC-FMO_2 structure is colored yellow. Contour levels for the RC-FMO_1 cryo-EM map and the *Hb*RC 2*Fo*-*Fc* electron density map are 2.4σ and 1.2σ, respectively.

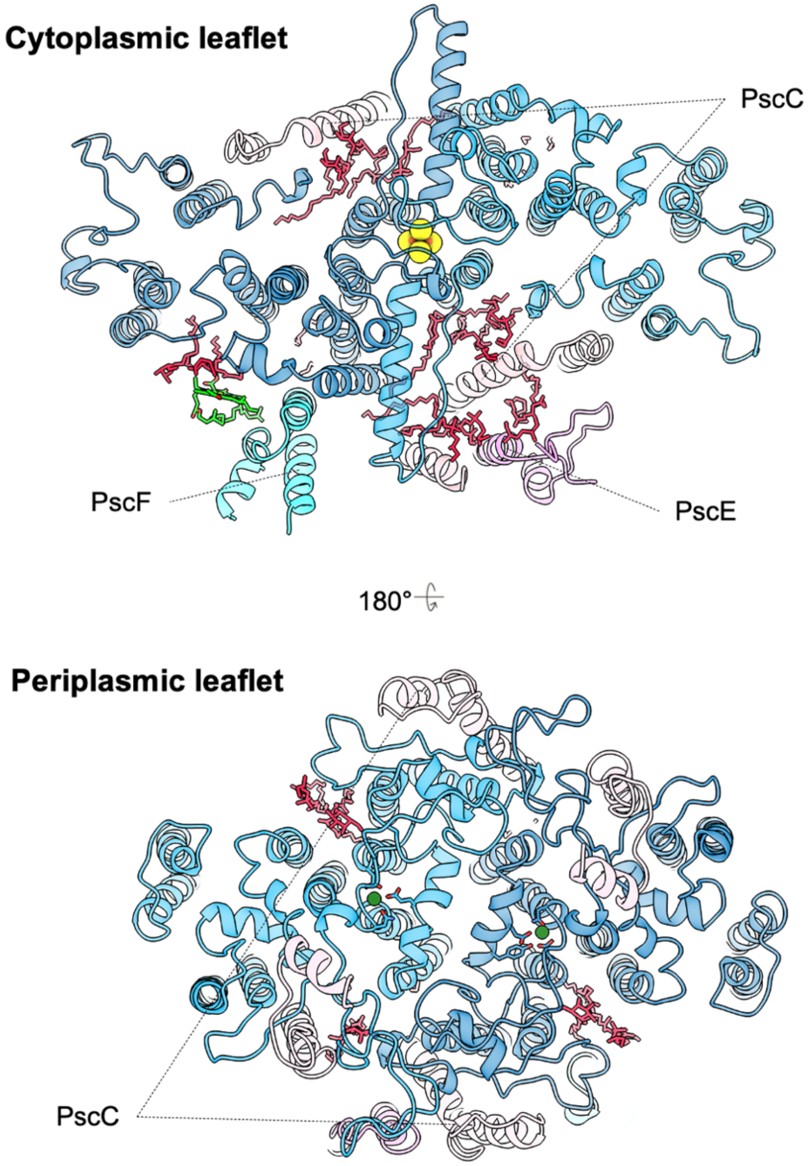

Supplementary Fig. 13 | Membrane lipids play an important role in maintaining the integrity of the photosynthetic assembly. Color codes: PscA1 – blue; PscA2 – light blue; and PscC - pink. Lipids in cytoplasmic (upper) and periplasmic leaflets (lower) are colored in crimson. Lipids in the cytoplasmic leaflet are sandwiched between PscA, PscC, and PscE. Linker BChl is colored in light green.

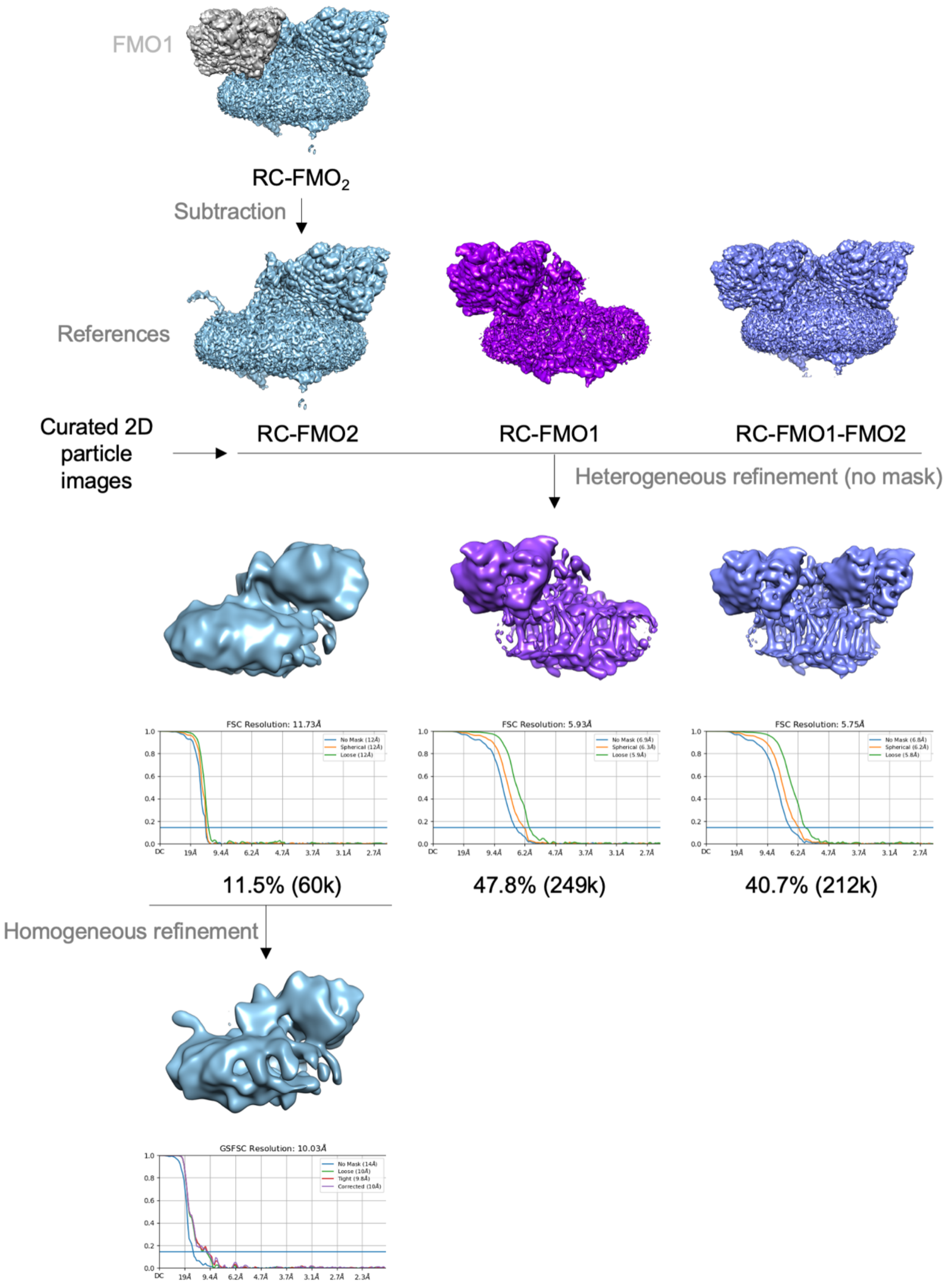

Supplementary Fig. 14 | Supervised 3D classification or heterogeneous refinement using the densities of RC-FMO1, RC-FMO2, and RC-FMO1-FMO2. The density of RC-FMO2 (light blue) was generated by the subtraction of FMO1 density (grey) from the RC-FMO1-FMO2 density (trendy blue). The RC-FMO1 density is shown in color purple.

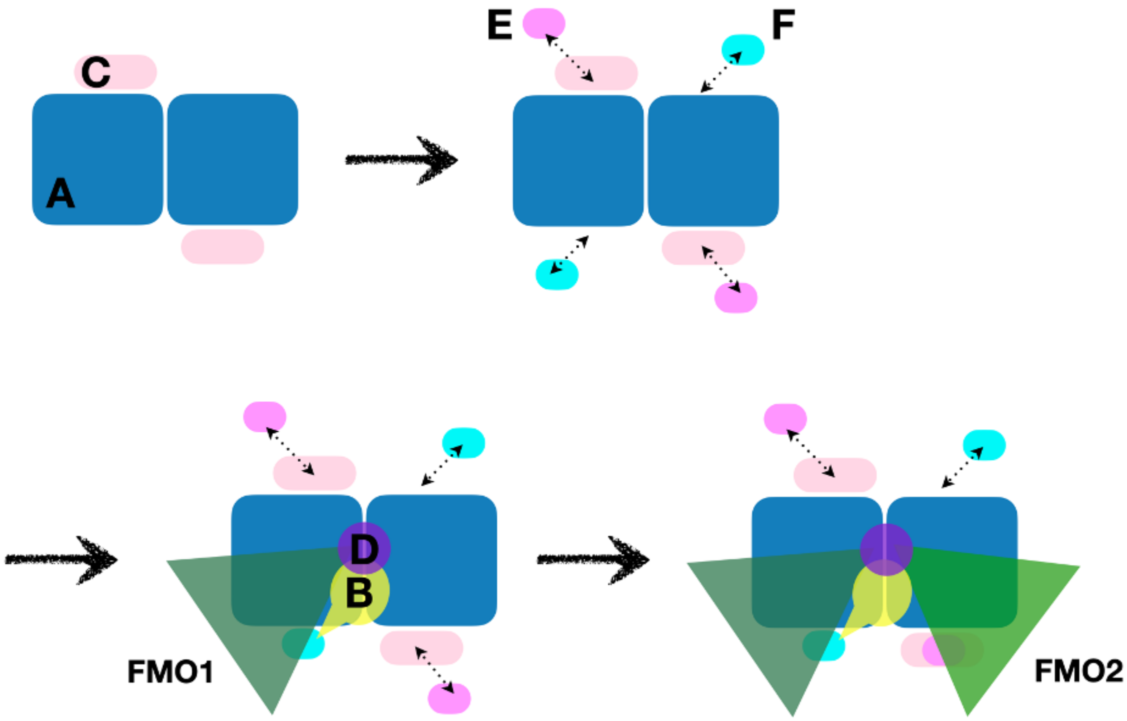

Supplementary Fig. 15 | Proposed model for GsbRC-FMO$_2$ supercomplex assembly process. Color codes: FMO1 – dark green; FMO2 – forest green; PscA1 – blue; PscA2 – light blue; PscB – yellow; PscC – light pink; PscD – purple; PscE – magenta; and PscF - cyan. Arrows indicate the assembling sequence.

SUPPLEMENTARY TABLES

Supplementary Table 1. Mass spectrometry results of *Chlorobaculum tepidum* RC-FMO sample from the native-gel electrophoretic bands.

Accession number	Protein name	Description	Sum PEP score	Coverage (%)	# unique peptides	# AAs	MW (kDa)	Abundance 1	Abundance 2	Abundance 3
Q46393	FMO	Bacteriochlorophyll a protein OS=Chlorobaculum tepidum (strain ATCC 49652 / DSM 12025 / NBRC 103806 / TLS) OX=194439 GN=fmoA PE=1 SV=3	514	83	52	366	40.3	2.0E+10	2.5E+10	1.8E+10
Q8KAY0	PscA	Photosystem P840 reaction center, large subunit OS=Chlorobaculum tepidum (strain ATCC 49652 / DSM 12025 / NBRC 103806 / TLS) OX=194439 GN=CT2020 PE=1 SV=1	201	30	36	731	81.7	3.0E+9	4.0E+9	2.9E+9
Q8KAY1	PscB	Photosystem P840 reaction center, iron-sulfur protein OS=Chlorobaculum tepidum (strain ATCC 49652 / DSM 12025 / NBRC 103806 / TLS) OX=194439 GN=pscB PE=1 SV=1	25	25	7	231	23.5	6.6E+8	9.3E+8	6.1E+8
O07091	PscC	Cytochrome c OS=Chlorobaculum tepidum (strain ATCC 49652 / DSM 12025 / NBRC 103806 / TLS) OX=194439 GN=pscC PE=1 SV=1	185	41	17	206	22.7	2.0E+9	2.4E+9	1.7E+9
Q8KEP5	PscD	P840 reaction center 17 kDa protein OS=Chlorobaculum tepidum (strain ATCC 49652 / DSM 12025 / NBRC 103806 / TLS) OX=194439 GN=pscD PE=1 SV=1	165	78	28	143	16.6	1.2E+9	1.8E+9	1.2E+9
Q8KDI3		Uncharacterized protein OS=Chlorobaculum tepidum (strain ATCC 49652 / DMS 12025 / NBRC 103806 / TLS) OX=194439 GN=CT1067 PE=4 SV=2	7	58	3	59	6.7	1.0E+6	1.0E+6	7.9E+5
Q8KG87	Ric1	Ric1 protein OS=Chlorobaculum tepidum (strain ATCC 49652 / DSM 12025 / NBRC 103806 / TLS) OX=194439 GN=CT0081 PE=3 SV=1	34	28	2	58	6.2	6.7E+7	7.8E+7	5.8E+7

Supplementary Table 2. Mass spectrometry results of *Chlorobaculum tepidum* RC-FMO sample from the SDS-PAGE bands with the sizes less than 15 kDa.

Accession number	Protein name	Description	Sum PEP score	Coverage (%)	# unique peptides	# AAs	MW (kDa)	Abundance 1 (< 15 kDa / > 15 kDa)	Abundance 2 (< 15 kDa / > 15 kDa)	Abundance (blank; control)
Q8KDI3		Uncharacterized protein OS=Chlorobaculum tepidum (strain ATCC 49652 / DMS 12025 / NBRC 103806 / TLS) OX=194439 GN=CT1067 PE=4 SV=2	15.5	58	5	59	6.7	2.09E+6 / 3.63E+5	2.44E+6 / 9.12E+4	0
Q8KG87	Ric1	Ric1 protein OS=Chlorobaculum tepidum (strain ATCC 49652 / DSM 12025 / NBRC 103806 / TLS) OX=194439 GN=CT0081 PE=3 SV=1	4.8	26	1	58	6.2	5.27E+6 / 1.85E+5	3.47E+6 / 1.24E+5	0

Supplementary Table 3. Amino acid composition of the PscE subunit.

Amino acid	Count	Percentage (%)
Alanine	4	6.8
Arginine	2	3.4
Asparagine	0	0.0
Aspartate	2	3.4
Cysteine	1	1.7
Glutamine	1	1.7
Glutamate	8	13.6
Glycine	3	5.1
Histidine	0	0.0
Isoleucine	3	5.1
Leucine	5	8.5
Lysine	11	18.6
Methionine	3	5.1
Phenylalanine	0	0.0
Proline	3	5.1
Serine	4	6.8
Threonine	3	5.1
Tryptophan	1	1.7
Tyrosine	1	1.7
Valine	4	6.8

Supplementary Table 4. Cryo-EM data collection, refinement, and validation statistics of RC-FMO$_2$ and RC-FMO$_1$ assemblies of *Chlorobaculum tepidum*.

	RC-FMO$_2$ (EMDB: EMD-26471) (PDB: 7UEB)	RC-FMO$_1$ (EMDB: EMD-26469) (PDB: 7UEA)
**Data collection and processing**		
Magnification	47,259×	
Voltage (kV)	300	
Electron exposure (e^-/Å^2)	45.4	
Defocus range (μm)	-0.8 - -2.5	
Pixel size (Å)	1.04	
Symmetry imposed	C1	
Initial particle images (no.)	1,753,711	
Final particle images (no.)	157,486	142,020
Map resolution (Å)		
FSC threshold (0.143)	3.08	3.49
Map resolution range (Å)	2.74 – 3.45	3.01 – 3.87
**Model refinement**		
Initial model used (PDB code)	6M32	6M32
Model resolution (Å)		
FSC threshold (0.5 / 0.143)	3.3 / 3.0	3.8 / 3.4
Map sharpening *B* factor (Å^2)	-102.3	-136.6
Model composition		
Non-hydrogen atoms	37,570	26,313
Protein residues	4,006	2,793
Ligands		
BCL	73	48
GS0	2	2
G2O	4	4
SF4	3	3
Ca	2	2
F26	3	3
F39	3	2
Lipids		
LMG	6	7
LHG	10	6
B factors (Å^2)		
Protein	44.06	72.52
Ligands	44.10	74.81
RMS deviations		
Bond lengths (Å)	0.005	0.005
Bond angles (°)	0.906	0.932
Validation		
MolProbity score	1.66	1.51
Clashscore	5.37	4.64
Poor rotamers (%)	0.09	0.00
Ramachandran plot		
Favored (%)	94.62	96.06

Allowed (%)	5.31	3.90
Disallowed (%)	0.08	0.04
