# Supplementary material for: Molecular asymmetry of a photosynthetic supercomplex from green sulfur bacteria
Source: Nat Commun. 2022 Oct 3;13:5824. doi: 10.1038/s41467-022-33505-4 (PMC9529944; doi:10.1038/s41467-022-33505-4)
Supplement: Supplementary file 3 — Description of Additional Supplementary Files [file 41467_2022_33505_MOESM3_ESM.pdf]

Description of Additional Supplementary Files

File name: Supplementary Data 1

Description: Förster rates for all interfacial pigments in FMO and RC complexes.

File name: Supplementary Data 2

Description: *K* squared factors for pigments of FMO and RC complexes.
